# Transcriptional profile analysis of E3 ligase and hormone-related genes expressed during wheat grain development

**DOI:** 10.1186/1471-2229-12-35

**Published:** 2012-03-14

**Authors:** Delphine Capron, Said Mouzeyar, Aurélia Boulaflous, Christine Girousse, Camille Rustenholz, Christel Laugier, Etienne Paux, Mohamed Fouad Bouzidi

**Affiliations:** 1 Université Blaise Pascal, UMR 1095 GDEC, 24 avenue des Landais, F-63177 Aubière, France; 2 INRA, UMR 1095 GDEC, 234 avenue du Brézet, F-63100 Clermont-Ferrand, France

## Abstract

**Background:**

Wheat grains are an important source of food, stock feed and raw materials for industry, but current production levels cannot meet world needs. Elucidation of the molecular mechanisms underlying wheat grain development will contribute valuable information to improving wheat cultivation. One of the most important mechanisms implicated in plant developmental processes is the ubiquitin-proteasome system (UPS). Among the different roles of the UPS, it is clear that it is essential to hormone signaling. In particular, E3 ubiquitin ligases of the UPS have been shown to play critical roles in hormone perception and signal transduction.

**Results:**

A NimbleGen microarray containing 39,179 UniGenes was used to study the kinetics of gene expression during wheat grain development from the early stages of cell division to the mid-grain filling stage. By comparing 11 consecutive time-points, 9284 differentially expressed genes were identified and annotated during this study. A comparison of the temporal profiles of these genes revealed dynamic transcript accumulation profiles with major reprogramming events that occurred during the time intervals of 80-120 and 220-240°Cdays. The list of the genes expressed differentially during these transitions were identified and annotated. Emphasis was placed on E3 ligase and hormone-related genes. In total, 173 E3 ligase coding genes and 126 hormone-related genes were differentially expressed during the cell division and grain filling stages, with each family displaying a different expression profile.

**Conclusions:**

The differential expression of genes involved in the UPS and plant hormone pathways suggests that phytohormones and UPS crosstalk might play a critical role in the wheat grain developmental process. Some E3 ligase and hormone-related genes seem to be up- or down-regulated during the early and late stages of the grain development.

## Background

Wheat is one of the leading sources of plant proteins in the human diet, either directly or via its use as animal feed. The proteins are stored in the endosperm, which makes up about 80% of the mature grain. The size and composition of the endosperm will therefore largely determine the yield and quality of the wheat. In view of the rapid growth of the world's population, which is outpacing increases in crop yields, crop scientists are now being challenged to gain a clearer understanding of wheat grain development [1].

The grain is formed by a double fertilization that results in a diploid embryo and a triploid endosperm. Based primarily on endosperm development, three clearly defined wheat grain developmental phases have been identified. Firstly, the prestorage or cell division stage occurs in the primary endosperm cell, where free nuclear divisions take place to form the coenocyte. In wheat, this may contain over 2000 nuclei just 72 h after fertilization. Cellularization occurs over a period of about 24 h, followed by about 10 d of cell division and, to a lesser extent, cell expansion. Approximately 14 d after fertilization, cell division finally ceases, resulting in a total of 300,000 cells in the endosperm [2,3]. Grain filling starts with the deposition of starch and gluten proteins in endosperm cells for about 28 d, after which this process declines and the grain starts to desiccate. Physiologically, grain maturity is reached around 42 d after fertilization and the grain ripe for harvest some 1-2 weeks later. The weight of a mature grain is positively associated with the number of endosperm cells it contains [2,4,5].

Transition through the different stages of wheat grain development is finely regulated by gene expression. In plants, the ubiquitin-proteasome system (UPS) is one of the most important pathways for the control of gene expression [6]; for example, it accounts for nearly 6% of the Arabidopsis thaliana proteome [7]. Highly conserved among eukaryotes, the UPS participates in the control of signal transduction events through a selective degradation of regulatory proteins such as the transcription factors and cell cycle regulators that play an important role in controlling eukaryotic growth and development.

The UPS consists of the enzymatic activities required for the poly-ubiquitylation of target proteins, and then for proteolysis of these tagged proteins by the proteasome. This polyubiquitylation is achieved by the sequential actions of three enzymes: an E1 ubiquitinactivating enzyme, an E2 ubiquitin-conjugating enzyme, and an E3 ubiquitin ligase. E1 activates and links free ubiquitins that will be transferred to one of the E2 enzymes, while at the same time E3 ligase enzymes promote ubiquitylation by mediating a specific interaction between E2 enzymes and the target protein [8].

Based on their role in the UPS, and the large size of the ubiquitin ligase E3 gene family (more than 1000 in Arabidopsis), E3 ligases are the key factor that confers specificity on the degradation process. Each ubiquitin E3 ligase may act on the ubiquitylation of one or more specific target proteins [8,9].

Several types of E3 ubiquitin ligases have been described in eukaryotes and classified in terms of their mechanisms of action and subunit compositions. They act as either a monomeric protein (e.g. HECT (homology to E6-AP C terminus), RING (Really Interesting New Gene) and U-box) or as a multimeric complex (e.g. Cullin-RING Ligases (CRLs)). CRLs are subdivided into four groups that depend on the composition of the multi-subunit complexes: 1) the SCF complex (SkP1 (S-phase Kinase-associated Protein 1)-CulL1 (Cullin 1)-F-box), where F-box subunits are used to target specificity; 2) the Cul3-BTB complex (Cullin3-Bric a brac-Tramtrack-Broad), where BTB proteins carry a dual function to recognize targets and interact with Cul3; 3) the Cul4-DDB1 complex (Cullin 4-DNA Damage-Binding 1); and 4) the APC/C complex (Anaphase-Promoting Complex/Cyclosome) [8,10].

In Arabidopsis, more than 1000 members of the E3 ubiquitin ligase family have been predicted based on their sequences: 7 HECT, 477 RING, 64 U-box, more than 700 SCF, 80 BTB, 5 DDB1 and 10 APC/C [11]. Accordingly, in plants, the most widely represented E3 ligases are the SCF complexes and RING E3 ligases.

Most hormones are involved in multiple processes such as organ differentiation and development, and they impact each other through elaborate crosstalk strategies. By elucidating these hormone signaling pathways, it has become clear that the UPS plays an essential role in hormone perception and response [12-14]. The close relationships between the UPS and hormones were first described during the identification of an F-box protein called TIR1 (Transport Inhibitor Response 1), which acts as an auxin (AUX) receptor [15]. The F-boxTIR1 protein binds auxin to promote auxin-regulated transcription through the Ub-mediated degradation of AUX/IAA transcriptional regulators [16,17]. As well as auxin, other hormones have also been described as interacting with F-box proteins in order to regulate gene expression. Likewise, the F-box protein called COI1 (Coronatine insensitive1) serves as a receptor for jasmonic acid and targets JAZ (jasmonate ZIM-domain) proteins for degradation at hormone binding [18,19]. Hormones can control ligase activity either directly (as described above) or through the action of a hormone receptor. For example, gibberellin (GA) signaling involves the ubiquitylation of Della proteins by F-box proteins called SLEEPY1 (SLY1) or SNEEZY1 (SNE1), in combination with GA and the GID1 (GA-Insensitive Dwarf 1) receptor [20,21].

In addition to SCF complexes, many other types of E3 ubiquitin ligases have been linked directly to plant hormone signaling. For example, E3 Cul3-BTB ubiquitin ligases are implicated in the regulation of ethylene (ETH) biosynthesis [22]. Not surprisingly, several ubiquitin ligases have been linked to abscisic acid (ABA) responses. Two RING ligases, AIP2 (ABI3-Interacting Protein) and KEG (Keep On Going), promote normal ABA signaling by regulating the abundance of ABA-responsive transcription factors, namely ABI3 (ABA-insensitive3) and ABI5 (ABA-insensitive 5) [23,24]. Despite numerous examples in different signaling pathways, only a few E3 ligases encoded in plant genomes have been characterized.

The involvement of hormones in cereal grain development is now widely acknowledged. For instance, AUX and cytokinin (CK) levels rise shortly after fertilization [25]. CK in the grains and roots during the early phase of rice grain development also play an important role in regulating grain filling patterns, and consequently the grain filling percentage [26]. Furthermore, a rise in the AUX content 9-11 d after pollination induces cellular differentiation events in maize endosperm [27]. The ABA level also rises during grain development as from 5 d after pollination, peaking 18 d after pollination [28]. GA is also involved in the release from dormancy of numerous species. In many cases, ABA antagonizes the effects of GA, with both events occurring at the level of gene expression. Finally, the ABA/GA equilibrium is a determining factor in signaling maturation during germination in maize [29].

This paper reports on a transcriptomic analysis with an emphasis for the first time on both E3 ligase coding genes and hormone-related genes at eleven different stages of wheat grain development (from 40 to 500°Cdays).

## Results

In 2010, Nadaud et al. [30] characterized the timing of the major phases of grain development in the Recital cultivar under the same experimental conditions used in this study. Briefly, the rapid grain dry mass accumulation rate peaked at about 255°Cdays after anthesis and then decreased; 95% of the final grain dry mass was set at about 760°Cdays. A rapid accumulation of water in the grains started immediately after anthesis and a plateau was reached between 271°Cdays and 650°Cdays; then the grains started to dehydrate. Cell multiplication in the endosperm reached its maximum rate at 100°Cdays and stopped at about 270°Cdays. Cell multiplication was concomitant with the start of the water plateau and the slowdown of dry mass accumulation. The present study concerned the early stages of grain development (40 to 500°Cdays), thus covering the whole cell division stage and the first half of the grain filling stage.

### Genes expressed differentially during wheat grain development

**Figure 1:**
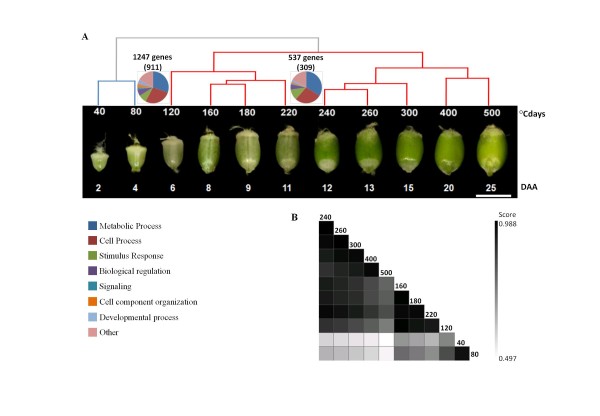
**Clustering of 9284 differentially expressed genes (DEGs)**. (**A**) Hierarchical clustering of two biological replicate samples of wheat grain development. The medians of twelve expression values were used to cluster the samples (see Methods). Stages are shown as °Cdays and days after anthesis (DAA). Scale bar: 0.5 cm. A paired *t*-test analysis (p < 0.01, adjusted Bonferroni correction) was performed to identify the genes whose expression levels changed significantly during the transitions between [40-80] and [120-500° Cdays]. Pie chart classifications for biological processes were inferred from various sources (see Methods). Unclassified UniGenes were omitted from the pie charts: the number of GO terms is shown in bracket next to the chart. (**B**) Pearson's correlation coefficient matrix of the clusters in panel (**A**).

To identify the genes involved in the early stages of wheat grain development, a NimbleGen microarray analysis (with 39,179 UniGenes) was performed (ref. A-MEXP-1928). This microarray was hybridized with wheat grain cDNAs generated at early developmental stages starting approximately 2 d after anthesis (40°Cdays under our experimental conditions), and including the stages corresponding to a broad time-span of grain filling (up to 500°Cdays). As shown in Figure 1A, a total of eleven stages of grain development were screened. A LIMMA analysis of two biological replicates and a dye-swap replicate resulted in the identification of 9284 differentially expressed genes (DEGs; p < 0.01) during the eleven developmental stages (see Additional file 1).

A cluster analysis of grain developmental stages using Pearson's correlation coefficient between DEGs revealed two large clusters with a correlation coefficient that was more than 0.95 within each cluster but less than 0.85 between clusters (Figure 1B). The first cluster comprised the two early stages (40 and 80°Cdays), and was clearly separated from the second cluster comprising the remaining stages (120 to 500°Cdays) (Figure 1A). The second cluster could be divided into two subgroups comprising the 120 to 220°Cdays stage and the 240 to 500°Cdays stage. These results indicate that significant changes in the expression profiles of genes during wheat grain development occur at the transitions between 80 to 120°Cdays and 220 to 240°Cdays. A paired *t*-test analysis (p < 0.01, adjusted Bonferroni correction) was performed to identify the genes whose expression levels changed significantly during these transitions. Subsequently, 1247 genes were found to be differentially expressed between [40-80°Cdays] and [120-500°Cdays] and 537 between [120-220°Cdays] and [240-500°Cdays].

To identify the biological processes involved in these transitions, we analyzed the Gene Ontology terms (GO terms) of the differentially expressed genes (1247 and 537) using the blast2GO package [31]. The GO terms obtained using the annotation procedure were mapped to a plant-specific GOslim in order to generate a more concise annotation. The level 2 GOslim classification of annotated UniGene sequences is reported in Figure 1. Among the annotated sequences, 911 were attributable to the transition between 80 and 120°Cdays and 309 to the transition between 220 and 240°Cdays. For the remaining 564 (31.6% of the total), no information on the plant tissue or organ source was available among the features of the database entries. The terms most widely represented in the two transition periods were mainly associated with metabolic and cellular processes. Nevertheless, during the first transition between 80 to 120°Cdays, UniGene sequences were mainly associated with the cellular component organization and developmental processes, while during the second transition between 220 and 240°Cdays, UniGene sequences were mainly associated with the response to stimulus. Using the fatiGO program [32], we extracted the terms that were found to be enriched among the DEGs associated with each transition with respect to the rest of the array. Details of this enrichment are listed in Additional file 2. During the 80-120°Cdays transition, enriched GO terms are involved in the defense response (e.g. serine-type endopeptidase inhibitor activity) and with storage proteins (e.g. nutrient reservoir activity). By contrast, no enriched GO terms were found in the DEGs during the 220-240°Cdays transition (see Additional file 2).

**Figure 2:**
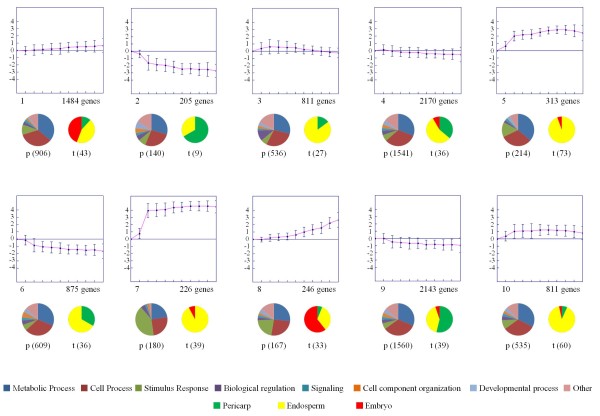
**Patterns of gene expression during wheat grain development**. Differentially expressed genes across 11 time-points of wheat grain development were grouped into 10 clusters using the K-means clustering algorithm. The ratio value (on the log_2 _scale) of expression is on the y-axis and the time of development in °Cdays is on the x-axis. The curve represents the mean of the expression ratio (on the log_2 _scale) in each cluster, and error bars represent the SD. Tick marks on the x-axis represent the 11 developmental time-points at 40, 80, 120, 160, 180, 220, 240, 260, 300, 400 and 500°Cdays. Pie chart classifications for biological process (p) and tissue category (t) are represented under the cluster. Unclassified UniGenes were omitted from the pie charts: the numbers that were classified are shown in brackets under the chart.

To gain further insight into the expression profiles of DEGs, the 40°Cdays stage was used as a common reference to compare the expression of DEGs at subsequent stages. K-means clustering of the transformed data set was carried out with 10 as the prefixed number of groups (Figure 2). The number of genes grouped into each of the ten different clusters ranged from 205 (cluster 2) to 2170 (cluster 4). Based on mRNA accumulation trends, the ten K-mean clusters of gene expression patterns were further sorted into three classes: up-regulated (clusters 1, 5, 7, 8 and 10), down-regulated (clusters 2, 4, 6 and 9) and up-down-regulated (cluster 3). The majority of DEGs were down-regulated (5393 genes), followed by up-regulated genes (3080 genes) and up-down-regulated genes (811 genes). Changes to the magnitude of gene expression levels during wheat grain development were not uniform, and the greatest changes were found in clusters 2, 5 and 7. Genes in clusters 5 and 10 were up-regulated during all the stages studied, i.e. from 40 to 500°Cdays, with expression peaks at 260 and 220°Cdays, respectively. The decrease in the expression level of genes in clusters 2 and 6 started at the early stages of wheat grain development, i.e. at 40°Cdays, but this only occurred at 80°Cdays in cluster 9 and after a small increase in expression levels at 80°Cdays in cluster 4. In clusters 1 and 8, the increase in the expression level started at 80°Cdays (Figure 2).

By identifying similar sequences, the clusters identified here could be compared with those reported in other transcriptome analyses of developing wheat grain. In each study, grouping in a small number of gene clusters was dependent on the choice of the algorithm. Nevertheless, some trends were clear; for example, clusters 2, 3, 7 and 8 were very similar to clusters SOM2_4, SOM3_4, SOM3_1 and SOM1_1, respectively, described by Wan et al. [5], and clusters 1, 9, 7 and 6, respectively, described by Laudencia-Chingcuanco et al. [33] (see Additional file 3).

We also assigned putative locations of the main types of grain tissue (endosperm, embryo and pericarp) to the transcripts in each set clusters based on the assignment made by Wan et al. [5]. The details of putative locations are summarized in pie charts (Figure 2 and Additional file 4). The clusters 1, 4, 8 and 10 contain transcripts assigned to the three tissues (pericarp, embryo and endosperm). The clusters 2, 3 and 6 contain transcripts assigned only to the pericarp and the endosperm. The clusters 5 and 7 contain transcripts assigned to the embryo and the endosperm, with the majority of these transcripts related to the later.

In order to identify the biological processes involved in each of the ten different clusters, we analyzed the GO terms that label the differentially expressed genes. 6388 UniGenes (68.8% of the 9284) were finally annotated (see Additional file 5). The annotated GO terms in each cluster were fixed at level 2 (Figure 2). The most represented terms in each cluster were mainly associated with metabolic and cellular processes. Clusters 7 and 8 possessed a high level of UniGene sequences associated with stimulus response (see Additional file 5).

Using the fatiGO program, we extracted the GO terms that were found to be enriched among the DEGs associated with each cluster relative to the rest of the array. Details on the enrichment are listed (see Additional file 2). Analysis with Fisher's exact test (p < 0.05) showed that in cluster 3, numerous processes related to photosynthesis and protein synthesis were over-represented. In cluster 6, many processes related to chromatin organization, such as the nucleosome and nucleosome assembly, were over-represented, which could be related to cell division. And in clusters 5 and 7, numerous processes related to defense, storage protein synthesis and starch synthesis were over-represented (see Additional file 2).

### E3 ligase genes expressed differentially during wheat grain development

The 9284 DEGs identified during this study included genes involved in every aspect of cell metabolism, i.e. including both structural and regulatory genes. Among the latter, for example, were several genes encoding transcription factors or protein kinases. However, this study focused on the UPS, and more specifically on E3 ligase coding genes. Unfortunately, few E3 ligases have been characterized in cereals, and even fewer that are involved in grain development.

For this study, the well-documented sequence databases of E3 ligases from *Oryza sativa *and *A. thaliana *were used as queries to perform reciprocal BLASTN and BLASTX homology searches using the wheat UniGene set (see Materials and Methods). These analyses generated a final set containing 876 UniGenes potentially coding for E3 ligases in wheat (Table 1, Additional file 6). Among these 876 UniGenes, two classes of E3 ligases were found most frequently: E3 RING and E3 SCF, with respectively 302 and 456 genes. The other five categories (E3 U-box, E3 HECT, E3 APC/C, E3 Cul3-BTB, and E3 Cul4-DDB1) were less well represented (31, 9, 7, 65 and 6 genes, respectively) (Table 1).

**Table 1:** E3 ligase genes in wheat and differentially expressed genes (DEGs) identified during wheat grain development

E3 type	Total number in wheat UniGene set	DEGs number (%)
RING	302	69 (22.8)

U-box	31	8 (25.8)

HECT	9	2 (22.2)

APC	7	2 (28.6)

Cul4-DDB1	6	0 (0)

Cul3-BTB	65	13 (20)

SCF	456	79 (17.3)

Total	876	173 (19.7)

Details of each E3 family are presented in Additional file 6

Statistical analysis using LIMMA (p < 0.01) identified 173 potential E3 ligase genes that were differentially expressed, out of the 876 E3 ligase genes identified. As shown in Table 1, these 173 E3 ligases were then classified into families. Similarly, the most frequently represented families among the differentially expressed E3 ligases were E3 SCF and E3 RING, with 79 (17.3% of the total number of E3 SCF in the wheat UniGene set) and 69 (22.8% of the total number of E3 RING in the wheat UniGene set) DEGs, respectively. The other E3 ligase families (U-box, HECT, APC/C, and Cul3-BTB) were less well represented, although the percentages of DEGs for each were similar (Table 1). Interestingly, none of the six E3 Cul4-DDB1 genes was differentially expressed during wheat grain development.

**Figure 3:**
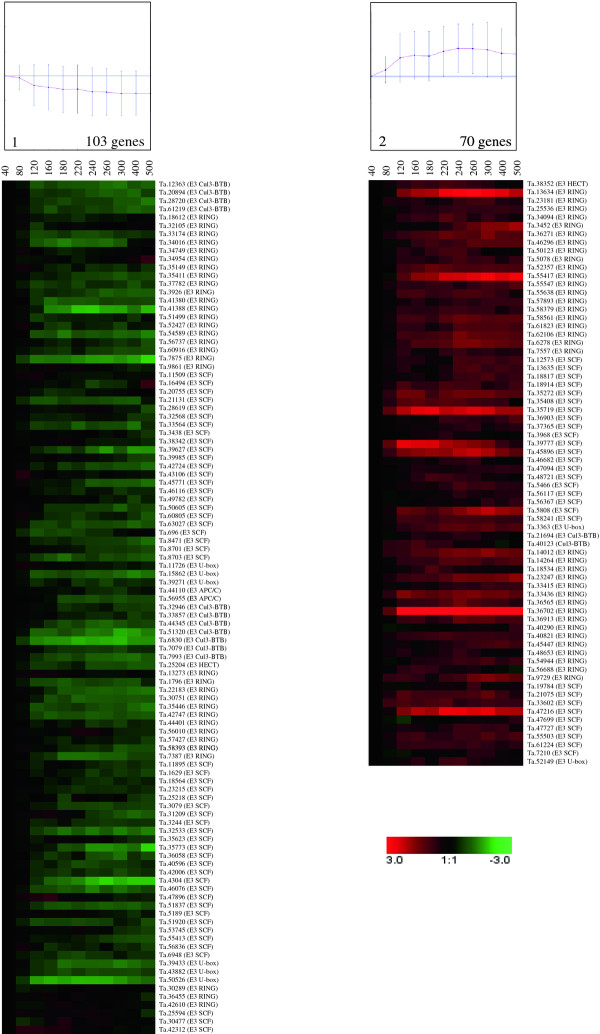
**Expression patterns of differentially expressed E3 ligase genes in developing wheat grain**. The differential expression profiles of E3 ligase coding genes at 11 time-points were grouped into two clusters (cluster 1: down-regulated genes; cluster 2: up-regulated genes) using the K-means clustering algorithm. The ratio value (on the log_2 _scale) of expression is on the y-axis and the time point of development in °Cdays is on the x-axis Among the 173 E3 ligases found to be differentially expressed in this study, 57 and seven had also been identified by Wan et al. [5] and Laudencia-Chingcuanco et al. [33], respectively. Moreover, six E3 ligases were identified in all three studies. The majority of these genes displayed similar average expression profiles in all the studies (see Additional file 3).

The 173 E3 ligase genes found to be differentially expressed shared different patterns during wheat grain development. Using a K-means clustering algorithm, the E3 ligase DEGs were grouped into two clusters (Figure 3): down-regulated genes (cluster 1) and up-regulated genes (cluster 2). Most of the E3 ligase genes were down-regulated (103 genes, cluster 1) when compared to the other cluster (70 up-regulated genes in cluster 2) (Figure 3). The genes in cluster 1 were down-regulated throughout the grain developmental process. The expression levels of these genes decreased from 80°Cdays to reach their lowest level at 300°Cdays; thereafter, the level of expression remained stably low. This cluster, which contained early specific grain genes, is composed of E3 SCF (50), E3 RING (33), E3 Cul3-BTB (11), E3 U-box (6), E3 HECT (1) and E3 APC/C (2). Cluster 2, containing up-regulated genes, is composed of E3 RING (36), E3 SCF (29), E3 Cul3-BTB (2), E3 HECT (1) and E3 U-box (2). The number of E3 Cul3-BTB and E3 SCF genes between the two clusters is not randomly distributed (see Additional file 7).

Among the potential E3 ligase genes identified, four shared homology with genes coding for proteins well known to be involved in hormone pathways (Table 2). The Ta.8471 gene is homologous with COI1 (F-box member of E3 SCF^COI1^) [19], the receptor for jasmonate (JA). The two genes Ta.23215 and Ta.21131 are homologous with TIR1 (F-box member of E3 SCF^TIR1/AFB1-3^) [16,17], the receptor for AUX, and the Ta.19784 gene presents significant homology with the EIN3-binding F-box protein (member of the E3 SCF^EBF1/EBF2 ^), which is involved in ethylene (ETH) signaling [34] (Table 2). The COI1 and TIR1 homologues, involved in JA and AUX signaling respectively, displayed maximum expression during the early stages of wheat grain development, while the F-box involved in ETH signaling showed increased expression during wheat grain development, reaching its maximum level at 260°Cdays.

**Table 2:** E3 SCF homologous genes differentially expressed during wheat grain development

Gene expression profiles are based on microarray results

### Hormone receptors and hormone-related genes during wheat grain development

Among the four homologous E3 ligase genes described above, three are known to be hormone receptors, which prompted us to search for hormone-related genes and their distribution during wheat grain development. During this study, the seven major plant hormone pathways investigated were ABA, AUX, BR, CK, ETH, GA and JA.

Genes coding for hormone-related genes from the *A. thaliana *and *O. sativa *databases were used as queries to search for their homologues in the wheat UniGene set, using the same approach as previously described for the wheat E3 ligases set. A homology search using reciprocal BLASTX and manual checking of the results identified 467 UniGenes that could be involved in the different hormone pathways in wheat (Table 3). Biosynthesis, signaling and response genes were identified (see Additional file 8). Analysis and classification of these 467 hormone-related genes revealed over-representation of the AUX pathway. The 133 AUX-related genes accounted for more than one-third of all hormone-related genes identified in the UniGene set (Table 3).

**Table 3:** Hormone-related genes and differentially expressed genes (DEGs) during wheat grain development

Hormone-related genes	Total number in wheat UniGene set	DEGs number (%)
Auxin (Aux)	133	41 (30.8)

Brassinosteroid (BR)	29	9 (31.0)

Cytokinin (CK)	47	17 (36.2)

Gibberellic acid (GA)	67	18 (26.9)

Ethylene (ETH)	83	15 (18.1)

Abscisic acid (ABA)	53	18 (34.0)

Jasmonic acid (JA)	55	8 (14.5)

Total	467	126 (27.0)

Details of each hormone-related gene family are provided in Additional file 8

Of the 9284 DEGs identified by LIMMA analysis (p < 0.01), 126 of the 467 wheat hormone-related genes were differentially expressed during wheat grain development (Table 3). The three categories of genes (biosynthesis, signaling and response genes) were all represented in the list of differentially expressed genes. The largest number of DEGs concerned AUX-related genes that mainly coded for genes potentially involved in AUX signaling (26 genes implicated in AUX signaling, including two AUX receptors; see Additional file 8). The smallest class of differentially expressed hormone-related genes contained JA-related genes, of which only eight were differentially expressed during wheat grain development (Table 3).

**Figure 4:**
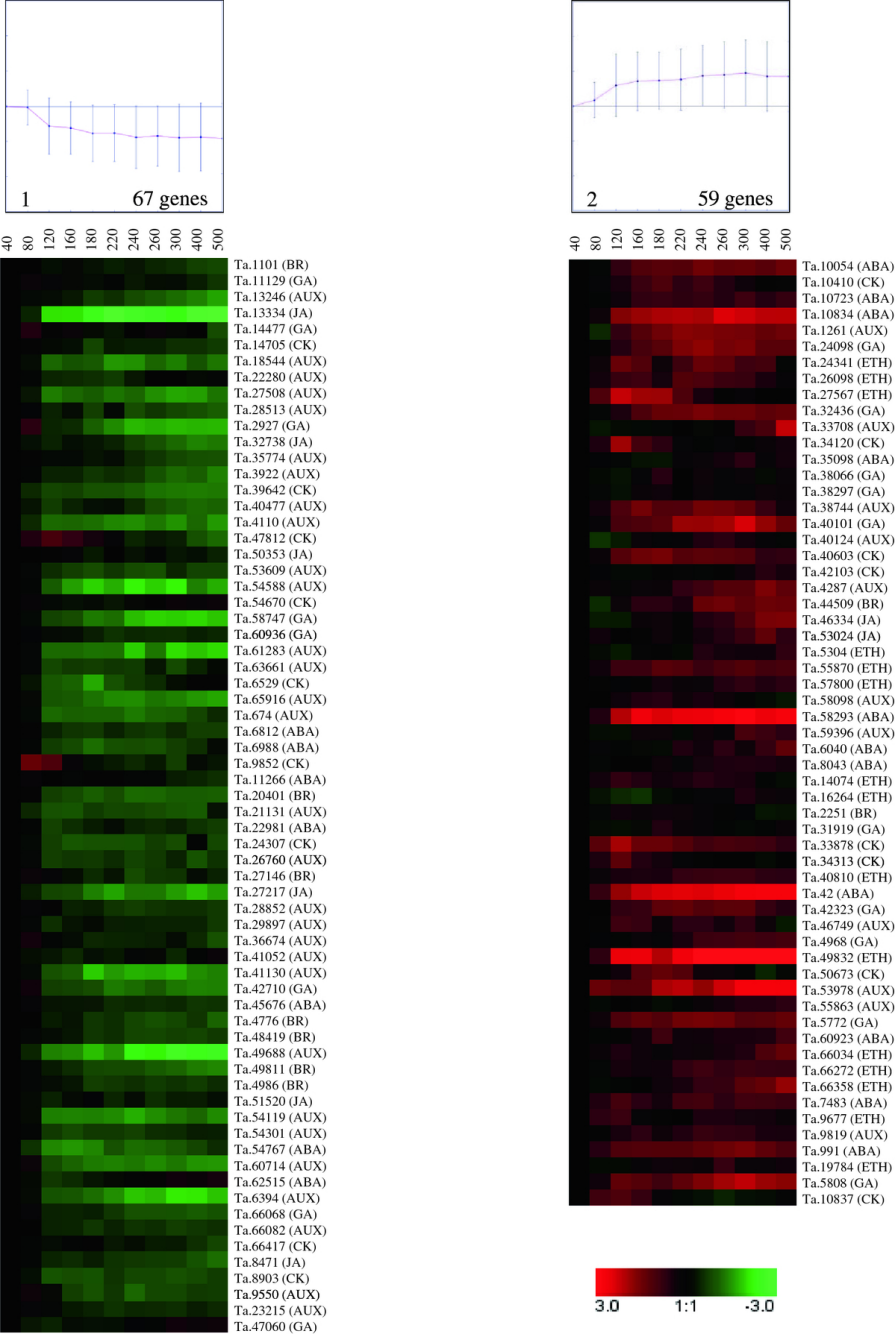
**Patterns of hormone-related genes expression in developing wheat grain**. The differential expression of genes at all 11 time-points were grouped into two clusters (cluster 1: down-regulated genes; cluster 2: up-regulated genes) using the K-means clustering algorithm. The ratio value (on the log_2 _scale) of expression is on the y-axis and the time of development in °Cdays is on the x-axis.

The 126 hormone-related DEGs were grouped into two clusters using the K-means clustering method (Figure 4). Cluster 1 (67 genes) grouped genes that were down-regulated during wheat grain development, and on average their profile declined between 80 and 500°Cdays. By contrast, cluster 2 (59 genes), grouped up-regulated genes, and this started at the earliest stage, i.e. from 40°Cdays. Cluster 1 contained 30 AUX-, seven BR-, six JA-, nine CK-, eight GA- and seven ABA-related genes. Cluster 2 contained eleven AUX-, two BR-, two JA-, eight CK-, ten GA-, eleven ABA- and the totality of ETH-related genes (15) (see Additional file 8). The number of Aux- and ETH-related genes between the two clusters is not randomly distributed (see Additional file 7).

Among the 126 hormone-related genes found to be differentially expressed during this study, 55 and 14 had been identified by Wan et al. [5] and Laudencia-Chingcuanco et al. [33], respectively. Moreover, six hormone-related genes were identified during all three studies. The majority of these genes displayed similar average expression profiles in all studies (see Additional file 3).

Among the hormone-related DEGs, several genes homologous with well-known hormone receptors were identified. The Ta.31919 gene is homologous with GID1 [35], the GA receptor. Two genes, Ta.39642 and Ta.8903, are homologous with the CK receptor CRE1/AHK [36,37]. Two additional genes, Ta.49832 and Ta.66358, displayed strong homology with ETR1 and EIN4, respectively, which are both ETH receptors [38-40] (Table 4). The expression level of the GA-receptor homologue, Ta.31919, reached a maximum at 180°Cdays and then declined (Table 4). The two CK-receptor homologous genes were mostly expressed during the early stages of wheat grain development, and their expression decreased over time (Table 4). The two ETH-receptor homologous genes displayed similar expression patterns that increased towards the end of wheat grain development (Table 4).

**Table 4:** Hormone-receptor homologous genes differentially expressed during wheat grain development

Gene expression profiles are based on microarray results

### Validation of wheat microarray data

**Figure 5:**
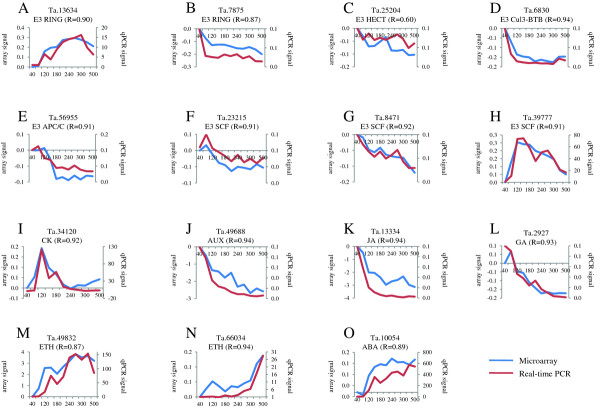
**Validation of array data by comparing the expression estimates from NimbleGen wheat microarrays and quantitative reverse transcription PCR for differentially expressed genes**. (A) - (H): Differentially expressed E3 ligase genes. (I) - (O): Differentially expressed hormone-related genes. Blue graphs represent DNA microarray data depicting the expression intensity of each transcript (left y-axis); red graphs depict quantitative reverse transcription RT-PCR results (right y-axis representing the relative expression level) during 11 developmental stages (x-axis representing the thermal time after anthesis (°Cdays)). The correlation coefficient (R) between the two graphs is indicated for each gene. Values for the two curves represent the ratio between the value for each stage and the 40°Cday value.

To assess the reliability of microarray data, quantitative reverse transcription PCR (qRT-PCR) was used to measure the expression level of 26 selected genes (Figure 5 and Additional file 9). A minimum of one gene from each E3 ligase family (Figure 5A-H) and from each hormone family, were selected (Figure 5I-O). RNAs isolated from a third biological sample (different from the set of biological samples used for hybridization on the array) were used as a template for amplification. The Pearson correlation coefficient for each gene was calculated between the wheat microarray data and qRT-PCR data. The relative expression levels determined by qRT-PCR revealed excellent concordance with those determined using the arrays, with correlation coefficients ranging from 0.81 to 0.97 in 22 out of the 26 cases, while the other four varied between 0.79 and 0.39. Given that the same variations in expression profiles were observed with both qRT-PCR and microarray analyses, the microarray results were deemed to be reliable.

## Discussion

Molecular development programs in plants are highly complex and require the intervention of several metabolic pathways, the activities of which need to be finely controlled and coordinated. Given that grain number and grain size are the major components of wheat yield, the grain has been the subject of numerous ecophysiological, genetic and molecular studies.

In the absence of sufficient wheat genome sequence data, expressed sequence tag and transcript assembly sequences representing the transcriptome have been used as foundations for the development of wheat oligonucleotide microarrays for large-scale gene expression profiling studies (e.g. [5,41-48]). However, some crucial and important signaling pathways have never been explored in wheat in general, and in the developing grain in particular. In order to study the expression profiles of these pathways during grain development, a NimbleGen microarray representing 39,179 UniGenes was used.

Cell division and grain filling are the main biological processes that determine the size and ultimate fate of the grain [49]. To identify some of the pathways involved in these crucial processes, samples collected at eleven developing grain stages were used, starting very early after anthesis (i.e. 40°Cdays) and covering the cell division and grain filling stages. In this way, the DEGs obtained at these developmental stages could reflect actual grain development, and provide useful information to clarify the molecular mechanisms underlying endosperm development.

During this study, of the 39,179 UniGenes represented on the microarray, 9284 UniGenes were differentially expressed during wheat grain development. From this set of 9284 DEGs, two large clusters of developmental stages were identified (Figure 1: cluster 1 from 40 to 80°Cdays and cluster 2 from 120 to 500°Cdays). Two additional subclusters were identified within the latter. This analysis revealed substantial differences in the whole wheat transcriptome during the transition from 80 to 120°Cdays. This finding agreed with the observations made at 6 d after anthesis by Drea et al. [50], who reported that the peripheral endosperm begins to cellularize at that time point, and that other cell types within the endosperm start to differentiate. Similarly, Rogers and Quatrano [4]divided wheat grain development into five major stages, from I to V. Based on their observations, the 40 to 80°Cdays stage in the present study corresponded to stage I, during which fertilization and a coenocytic endosperm occur. The transition between 220 and 240°Cdays corresponds to the transition from stage II (cellularization and early grain filling) to stage III (grain filling) [4]. This transition from the cell division (stage I and II) to the storage phase of endosperm development (stage III) was previously observed as being accompanied by an extensive reprogramming of gene expression patterns [5,33,50,51]. A recent study [30] showed that the end of endosperm cell multiplication (indicating the end of the cell division stage) in the Recital cultivar was observed at around 270°Cdays. This was shown to be concomitant with the beginning of the water plateau and a slowdown of dry matter accumulation in the grain. This developmental time overlapped with the start of endosperm cell expansion and filling [30]. The clustering based on molecular analysis in our study thus fitted well with the developmental stages described previously.

A *t*-test analysis (p < 0.01) was made between these clusters and revealed 1247 DEGs during the [40-80°Cdays] to [120-500°Cdays] transition and 537 DEGs during the [120-220°Cdays] to [240-500°Cdays] transition. The larger number of DEGs found during the first transition may indicate that substantial changes occur during this phase, compared to the second transition. An analysis using Fisher's exact test (p < 0.05) on the two sets showed that the first transition is enriched in two GO terms related to the defense response (e.g. serine-type endopeptidase inhibitor activity) and with storage proteins (e.g. nutrient reservoir activity). This indicates that as early as 2 days after anthesis, marked transcriptional activity dedicated to the synthesis of nutrient components (storage proteins) occurs, together with the transcription of genes coding for protease-inhibitors and alpha-amylase inhibitors intended to protect these two components from degradation by different hydrolases. This finding is consistent with that of Yang et al., who identified these gene families as wheat seed-specific genes [52]. No enriched biological process was observed at the 240-260°Cdays transition.

The ten clusters identified here were compared with those reported following other transcriptome analyses of developing wheat grain [5,33] (see Additional file 3). Clusters with similarities in terms of sequence composition also displayed similar average expression profiles.

According to the enrichment analysis of the ten clusters, those made up of genes mainly expressed during the cell division stage were enriched in photosynthesis (cluster 3), chromatin organization and nucleotide metabolism genes (cluster 6). Moreover, genes involved in chromatin organization were mainly expressed at 40 and 80°Cdays, when endoreplication level is at its peak. By contrast, clusters comprising genes mainly expressed during the grain filling stage (clusters 5, 7, 8 and 10) were enriched in defense, starch synthesis and/or storage protein genes. Interestingly, genes involved in the defense process encode a serine-type endopeptidase inhibitor, alpha-amylase inhibitor. These genes are protease inhibitors and protect cereal seed from attack by endogenous hydrolase. Thus, during the grain filling stage, in addition to the transcript accumulation of genes encoding storage proteins, genes involved in protecting the main nutritional resource of the developing seed from both endogenous and exogenous degradation are also active [52]. This phenomenon therefore appears to be important as it constitutes the significant change seen during the first transition (see above).

The relative distribution of the transcripts between the different grain tissues (pericarp, embryo and endosperm) is similar to the distribution described by Wan et al. [5]. For example, the clusters 1 and 8 contain the majority of the transcripts related to the embryo with an increasing expression profile during the development of the grain. This is in accordance with the description of the clusters SOM2_1 and SOM1_1 respectively of Wan et al. [5]. Similarly, the cluster 5 and 7 contain endosperm-related transcripts. Interestingly, the cluster 5 is enriched in GO terms related to defense and starch synthesis, while the cluster 7 is enriched in GO terms related to defense and storage protein synthesis, confirming their belonging the endosperm tissues. The overall distribution of the DEGs between the grain tissues is similar to the one described by Wan et al. [5].

The comparison of the expression profiles and the tissue distribution of the transcripts show some conservation of effects across the conditions, transcriptome platforms (Affymetrix vs. Nimblegen) and wheat cultivars used (we used Recital; Laudencia-Chingcuanco et al. [33] used Bobwhite, and Wan et al. [5] used Hereward).

However, the gene expression profiles analyzed during this study may reflect the expression of the three homeologous genes that may be found in the hexaploid wheat genome. Even though a NimbleGen microarray is used to discriminate homeologous genes in polyploid species [53], the microarray chip used during this study was not designed to distinguish wheat homeologous genes. In addition, the UniGene set used in this study was designed using automatic processes for the assembly and building of contigs, so it could not distinguish homeologous genes. The 60-mer probes were used to ensure overall high gene specificity, but this was at the expense of discrimination between homeologous genes. Therefore, differences between homeologous genes may exist and this problem needs to be addressed by using specific probes or homeologous-specific primers in qRT-PCR analysis.

### E3 ligase involvement in wheat grain development

As the wheat genome has not yet been fully sequenced, global analysis of the 9284 UniGenes expressed differentially in terms of gene family enrichment was certainly partial. However, the identification of genes important to wheat grain development could be carried out by analyzing some particular pathways. In this respect, previous studies had involved a global analysis of wheat microarray data and reported the identification of DEGs belonging to certain families of genes, such as transcription factors [5,33,45]. In addition, recent studies have underlined the importance of the cell cycle, phytohormone signaling, carbohydrate supply and the ubiquitin pathway to grain yield [26,27,54]. We therefore focused our analysis on the UPS pathway, which is increasingly recognized as a major component in a growing number of processes in plants. Several protein families are involved in the UPS pathway; among these proteins, the E3 ligase proteins provide specificity to the reaction.

For the first time in wheat, this study thus reports an inventory of E3 ligase coding genes. Among the 39,179 UniGenes, 876 E3 ligase coding genes were identified. During their study, Du et al. identified 1332 and 1305 E3 ligases in rice and *A. thaliana*, respectively [11]. Even though the precise number of E3 ligase coding genes in the wheat genome remains undetermined, our finding is probably an underestimation, and may only account for about half of the total number of this class of genes in wheat. However, by comparing our results and those obtained by Du et al. [11], we observed a similar relative number of genes coding for each E3 ligase family. These E3 ligases are classified in seven families (E3 RING, E3 SCF, E3 U-box, E3 HECT, E3 APC/C, E3 Cul4-DDB1, and E3 Cul3-BTB), among which the E3 SCF and E3 RING ligase genes are over-represented. This relative distribution of the different E3 ligases in each family seems to have been conserved in different plant species [11]. Of these 876 E3 ligase genes, 173 were identified as being differentially expressed. The large number of E3 ligases differentially expressed in the developing wheat grain (19.7% of all E3 ligase genes) may indicate the importance of E3 ligases to the different processes that occur during wheat grain development. In this context, the involvement of some E3 SCF and E3 RING ligases has already been suggested in *Arabidopsis*, maize, and rice [54-56]. For instance, Jain et al. [57] identified 31 F-box coding genes that were differentially expressed between five stages of rice grain development. As observed by Laudencia-Chingcuanco et al. [33] during a transcriptional study of wheat grain, genes highly expressed at a particular stage may indicate their relevance to stage-specific developmental functions. This may therefore suggest diverse roles for these E3 ligases during the different processes that occur during wheat grain development.

Classification of the 173 differentially expressed E3 ligases as a function of their expression profiles showed that E3 ligase gene families were not equally distributed between the two expression clusters (Figure 3). A majority of E3 SCF and E3 Cul3-BTB coding genes seemed to be significantly activated during the early stages of wheat grain development analyzed in this study (see Additional file 7). This finding may indicate the active involvement of these classes of E3 ligases during the cell division stage of wheat grain development. However, it is necessary to determine in other model plants, such as rice, whether enrichment of these E3 ligase families was coincidental or reflected a true distribution pattern.

Interestingly, the E3 APC/C genes studied displayed the highest level of expression at the beginning of wheat grain development, when cell multiplication is at its maximum. E3 APC/C ligases are known for their involvement in cell cycle regulation [58], and Fu et al.

[59] recently found that E3 APC/C genes are positively associated with grain yield in maize. Even if the number of E3 APC/C ligase coding genes identified in this study is too small, the trends observed and previous studies [58,59] seem to indicate that these genes are implicated during the cell division stage early on in wheat grain development.

Four E3 SCF subunits (F-box) were particularly interesting because they share homology with the F-box proteins involved in hormone signaling in rice and *Arabidopsis*. These F-box proteins are implicated in the signaling of three major hormones, i.e. JA, AUX and ETH (Table 2). Among these four F-box proteins, three are actual hormone receptors. The involvement of E3 SCF and RING ligases in hormone signaling linked to plant development is well documented [14]. For instance, involvement of the E3 SCF complex, and its relationship with AUX in the cell division process during maize kernel development, has already been suggested by Liu et al. [55]. Accordingly, AUX, JA and ETH are likely to be implicated in wheat grain development, in collaboration with E3 ligases. However, this hypothesis needs to be tested directly by means of genetic or mutational experiments.

### Differentially expressed hormone-related genes during wheat grain development

By elucidating the hormone signaling pathway, it is clear that the UPS plays an essential role in hormone perception and responses. Several E3 ligases have been fully characterized during plant development in connection with hormones [14]. Several studies have focused on the assay of hormones in wheat grains grown under either optimal or stressed conditions such as drought and waterlogging [60,61]. However, little is known about the molecular involvement of these different hormones and their relation with E3 ligase in wheat grain development. For this reason, the expression of hormone-related genes during wheat grain development was monitored. Firstly, all AUX-, BR-, CK-, GA-, ETH-, ABA-, and JA-related genes were identified within the wheat UniGene set. By homology with hormone-related genes in rice and *Arabidopsis*, 467 genes involved in the different hormone pathways in wheat were determined. As mentioned above for E3 ligase coding genes, this number is probably an underestimation of the total number of hormone-related genes present in wheat.

Microarray analysis revealed 126 hormone-related DEGs during wheat grain development. Analysis of the expression profiles of these genes suggests the possibility of a specific distribution for each hormone during wheat grain development. Typically, two major groups were found: cluster 1, with genes down-regulated during the wheat grain development; and cluster 2, with genes up-regulated during the wheat grain development. The first cluster contained a majority of AUX-related genes (30 out of the 41 AUX-related genes). Among these AUX-related genes, the F-box proteins Ta.21131 and Ta.23215 which code for putative auxin-receptors are mainly expressed during the cell division (Table 2). This expression pattern could reflect the importance of AUX at the beginning of wheat grain development. Similar results have been reported in maize, where the involvement of AUX in cell division during kernel development has been suggested [55].

A second pattern corresponding to an increased level of expression during wheat grain development was also observed, and implied the considerable importance of these genes during grain filling or subsequent stages. This cluster contained all the ETH-related genes identified in the UniGene set and found to be differentially expressed; the majority of ABA-related genes and also some AUX-, BR-, GA-, JA-, and CK-related genes. Among these ETH-related genes, the expression of Ta.19884, an EIN3-binding F-box, raised during the wheat grain development with a maximum level at 260°Cdays (Table 2). Moreover, both the ethylene receptors ETR1 (Ta.49832) and EIN4 (Ta.66358) reached their maximum expression level at the late developmental stages used in this study (Table 4). In the other hand, ABA is known to be involved in both the synthesis of storage products and the desiccation and dormancy processes. Moreover, ABA levels are positively correlated with the grain filling rate at the early grain filling stage in wheat and rice [62,63]. This pattern of an increase in the expression levels of ABA-and ETH-related genes has also been found during maize kernel development [55]. The presence of ABA- and ETH-related genes at the grain filling stage may indicate the importance of both hormones at this stage in wheat, as is the case in rice and maize [55,62,63]. However, as well as these two important hormones, BR-, CK-, GA-, JA-and AUX-related genes may also play a role; not only in cell division, but also at the grain filling stage. Overall, the timing of the expression of hormone-responsive genes fits well with the accumulation of these hormones in wheat grain [60,61].

By comparing the timing of transcript accumulation of a collection of E3 ligase and hormone-related genes during wheat grain development, we have provided the first comprehensive analysis of the molecular involvement of these classes of genes in wheat. Some specific members of these large gene families were identified, and, in the future, they could be the subject of more specific studies to clarify their precise role in grain development. In addition, we observed a significant expression of E3 Cul3-BTB, E3 SCF and AUX-related genes during the early stages of the wheat grain development while ETH-related genes seemed preferentially expressed at the late stages used in this study. Nevertheless, these observations made during this work need to be tested by designing experiments to determine the particular involvement of each hormone; for example, using hormone synthesis or/and hormone transport inhibitors.

## Conclusions

Despite the importance of cereals as a major source of nutrition for humans, and the findings of several transcriptomic analyses, the molecular processes involved in wheat grain development are still poorly understood. This study describes the expression dynamics of global gene expression during early wheat grain development using NimbleGen microarrays, and its findings are consistent with those of previous studies. It also reports on a particular focus on E3 ligase genes and hormone-related genes. A comprehensive list of developmentally-regulated E3 ligase and hormone-related wheat genes, and their profiles of expression, was thus generated. These data may provide a useful framework to obtain new insights into the involvement of E3 ligase and hormone-related genes in wheat grain development.

## Methods

### Plant materials and growth conditions

Wheat (*Triticum aestivum *cv Recital) was grown from seed in a soil-based compost in a controlled-environment room under conditions of 16 h light and 8 h darkness at 19 ± 1°C and 45% relative humidity (RH). At the three-leaf stage, the seedlings were vernalized for 2 months under the growth conditions of short days (8 h photoperiod) and at a low temperature (4°C), after which they were placed in a greenhouse (17 ± 2°C night, 20 ± 2°C day, 16 h light at 450 Watts m^-2 ^and 45% RH). The first flowering spikelets of all tillers on each plant were tagged at anthesis and only grains from florets with the same anthesis date were harvested. Grains at 11 developmental stages were harvested at 40, 80, 120, 160, 180, 220, 240, 260, 300, 400 and 500 degree days (°Cdays). Frozen immediately in liquid nitrogen, the grains were then stored at -80°C to minimize the effects of wounding. The °Cdays are the sum of the average daily temperatures from anthesis to the day of harvest. The experiment was performed on three independent, grain sample pool replicates per developmental stage. For each developmental stage, three biological replicates were used, pooling grains from about 20 plants.

### Total RNA extraction

Total RNA for microarray and quantitative real-time PCR analyses were extracted from the grain sample pools using the method described by Bogorad et al. [64] with slight adjustments for small grain powder samples (0.5 g). An extraction was performed separately for each grain sample pool replicate. The RNA was then treated with DNaseI (Invitrogen). RNA integrity was assessed using the RNA 6000 Nano assay bioanalyzer (Agilent).

### Microarray design and analysis

Gene expression profiles were generated using two NimbleGen microarrays for wheat (ref. A-MEXP-1928) with two biological replicates for each developmental stage. Each microarray used comprised 39,179 UniGene (Wheat UniGene set v55) with three different probes per UniGene. Two sets of dye-swapped experiments were performed to obtain a total of four replicate hybridizations per developmental stage. cDNA was synthesized from total RNA using the SuperScript Double-stranded cDNA Synthesis Kit (Invitrogen) and labeled with fluorescent Cy3 and Cy5 dyes using a Two-Color DNA Labeling Kit (NimbleGen). Hybridization was performed using the NimbleGen Hybridization Kit, according to the manufacturer's instructions. Samples from the two biological replicates, labeled with the two different dyes, were randomly hybridized. Microarray images of each slide were obtained with an Innoscan 900 AL Microarray scanner (Innopsys) and read with the Mapix program (Innopsys).

The raw data files generated from all chips were imported to Nimblescan 2.5 software for detailed analysis. To stabilize variations in data from the chips, the raw data were normalized using the GC-RMA (Gene Chip Robust Multi Array Analysis) algorithm. The microarray results were filtered to select UniGenes differentially expressed by LIMMA [65] with a P-value < 0.01 using MeV 4_5_1 software http://www.tm4.org/mev/. The median of the 12 samples for each UniGene (three probes with two biological replicates and one dye-swap) was used for subsequent analyses. All the data have been submitted in MIAME-compliant form with the ArrayExpress database (Accession Number E-MTAB-690).

The developmental stages (experiments) of wheat grain were clustered using Pearson's correlation coefficient between the DEGs. To perform a clustering analysis of all the DEGs, the median of two biological replicates for each time-point was transformed into a comparison using 40°Cdays as the common reference point, and the DEGs were grouped using the K-means algorithm with a Euclidean distance based on log_2 _fold change data. All clustering operations were performed using the Genesis software [66].

### GO annotation using Blast2GO

The set of UniGenes grouped into each of the ten different clusters was functionally annotated by performing a Blast search with Blast2GO (http://www.blast2go.org) default parameters against the non-redundant (nr) protein sequence database [31]. Similarly, Blast2GO software v1.3.3 was used to obtain Gene ontology (GO) information from retrieved database matches. Finally, the 'Augment Annotation by ANNEX' function was used to refine annotations. GOslim 'goslim_plant.obo' was used to achieve specific GO terms by means of a plant-specific reduced version of the Gene Ontology. The output data of Blast2GO software were exported in text format, imported into Microsoft Excel spreadsheets, and used to generate pie charts (Figure 2). The hierarchical representation of the gene ontology was structured according to different levels, from the highest (level 1) parents corresponding to the three main GO categories (cellular component, biological process, molecular function) to the lowest, more specialized child terms (level 2, 3, 4, etc.). In the present study, GO annotated datasets were represented at level 2. This level was chosen because it greatly facilitates comparisons among sequence sets by highlighting the most significant differences (see also Additional file 5).

### GO enrichment

UniGenes belonging to each cluster were used to retrieve the corresponding probeset in the Wheat Affymetrix 61 K GeneChip^® ^http://www.affymetrix.com. The corresponding GOID for each probeset were then identified from the Blast2Go home page http://bioinfo.cipf.es/b2gfar/affychips:wheat and used for GO enrichment analysis with fatiGO ([32], http://fatigo.bioinfo.cnio.es). In this analysis, the wheat Affymetrix chip annotated by the Blast2Go team was used as a reference (annotated array) to perform Fisher's exact test. GO terms with a p-value less than 0.05 were considered to be significantly different and enriched in the cluster.

### Data retrieval

To identify E3 ligases and hormone-related genes homologous in wheat, the National Center for Biotechnology Information (NCBI, http://www.ncbi.nlm.nih.gov/blast/Blast.cgi), and The Institute for Genomic Research database (TIGR, http://www.tigr.org/db.shtml) resources were used. E3 ligases and hormone-related UniGenes were retrieved using all *Arabidopsis *and rice E3 ligase and hormone-related sequences as the query in a homology search with the BlastN, BlastX and TBlastX programs. The blast hits were filtered using an e-value threshold of 10-5 and an alignment length exceeding 80 bp. All sequences were checked for consistency and for the presence of specific protein signatures using the Interproscan program [67].

### Quantitative real-time PCR analysis

In order to validate microarray data on a few selected genes displaying differential expression patterns during wheat grain development, real-time PCR was performed using RNAs extracted from a third biological replicate of wheat grown under the conditions described above. The Ta2776 gene was used as the reference gene [68] and was amplified in parallel with the target gene to enable the normalization of gene expression. Real-time PCR products were detected using the iQ SYBR Green Supermix (Biorad) following the manufacturer's recommendations. Two micrograms of total RNA were reverse-transcribed into cDNA with oligodT using SuperScript II reverse transcriptase (Invitrogen) and treated with DNaseI (Invitrogen). Four microliters of a 40-fold dilution of this cDNA and 100 nM of each primer were used as a template for qRT-PCR. The PCR cycling conditions comprised an initial denaturation step of 95°C for 10 min, followed by 40 cycles of 95°C for 15 s and X°C for 60 s. The × temperature was identified beforehand by temperature gradient PCR in order to select the optimum temperature for each primer. The nucleotide sequence, × temperature (T_m_), amplicon size and efficiency of primers are listed in Additional file 10. A melting curve analysis was performed at the end of the PCR run over the range 55-95°C, increasing the temperature stepwise by 0.5°C every cycle. Baseline and threshold cycles (Ct) were determined automatically using Optical System Software (Biorad, France). Zero template controls were included for each primer pair, and each PCR reaction was performed in triplicate. The PCR efficiencies of target and reference genes were determined by generating standard curves. Relative expression values were calculated using the 2^-ΔΔCt ^method, with the 40°Cdays stage as a reference sample for ΔΔCt, according to the method described by Schmittgen and Livak [69].

## Competing interests

The authors declare that they have no competing interests.

## Authors' contributions

DC carried out RNA extractions, microarray hybridizations and real-time RT-PCR analyses, and drafted the manuscript. SM, AB, CG and MFB were involved in improving the manuscript. CR, CL and EP contributed to microarray design and production. SM and MFB supervised the project and finalized the paper. All authors read and approved the final version of the manuscript.

## Supplementary Material

Additional file 1 **Normalized data from wheat grain development microarray expression analysis. Data are presented as median values**. Click here for file

Additional file 2 **Information of enriched GO. GOID, term GO, process, p-value and probesets were listed in the file**. Click here for file

Additional file 3 **Number of common sequences (the whole set of DEGs, E3 ligase and Hormone-related genes) identified by sequence similarity between Affymetrix wheat targets (Wan et al., 2008)/EST (Laudencia-Chingcuanco et al., 2006) and the UniGene sequences identified in this study**. Click here for file

Additional file 4 **Putative tissue location of the transcripts DEGs**. Click here for file

Additional file 5 **Details of Blast2GO analysis**. Click here for file

Additional file 6 **Details of E3 ligase genes**. Click here for file

Additional file 7 **The relative distribution of the E3 ligase and hormone-related genes between cluster 1 (up-regulated genes) and cluster 2 (down-regulated genes)**. Click here for file

Additional file 8 **Details of hormone-related genes**. Click here for file

Additional file 9 **Validation of array data by comparing expression estimates obtained from NimbleGen wheat microarray and quantitative reverse transcription PCR for differentially expressed E3 ligase and hormone-related genes**. (A) -(H): E3 ligase genes. (I) -(K): Hormone-related genes. Blue graphs represent DNA microarray data depicting the expression intensity of each transcript (left y-axis); red graphs depict quantitative reverse transcription RT-PCR results (right y-axis representing the relative level of expression) at 11 developmental stages (x-axis representing the thermal time after anthesis (°Cdays)). The correlation coefficient (R) between the two graphs is indicated for each gene. Click here for file

Additional file 10 **Primer sequences used for qRT-PCR analysis**. Click here for file
