# Supplementary material for: Transcriptional profile analysis of E3 ligase and hormone-related genes expressed during wheat grain development
Source: BMC Plant Biol. 2012 Mar 14;12:35. doi: 10.1186/1471-2229-12-35 (PMC3405487; doi:10.1186/1471-2229-12-35)
Supplement: Additional file 9 — Validation of array data by comparing expression estimates obtained from NimbleGen wheat microarray and quantitative reverse transcription PCR for differentially expressed E3 ligase and hormone-related genes. (A) -(H): E3 ligase genes. (I) -(K): Hormone-related genes. Blue graphs represent DNA microarray data depicting the expression intensity of each transcript (left y-axis); red graphs depict quantitative reverse transcription RT-PCR results (right y-axis representing the relative level of expression) at 11 developmental stages (x-axis representing the thermal time after anthesis (°Cdays)). The correlation coefficient (R) between the two graphs is indicated for each gene. [file 1471-2229-12-35-S9.PPT]

## Slide 1
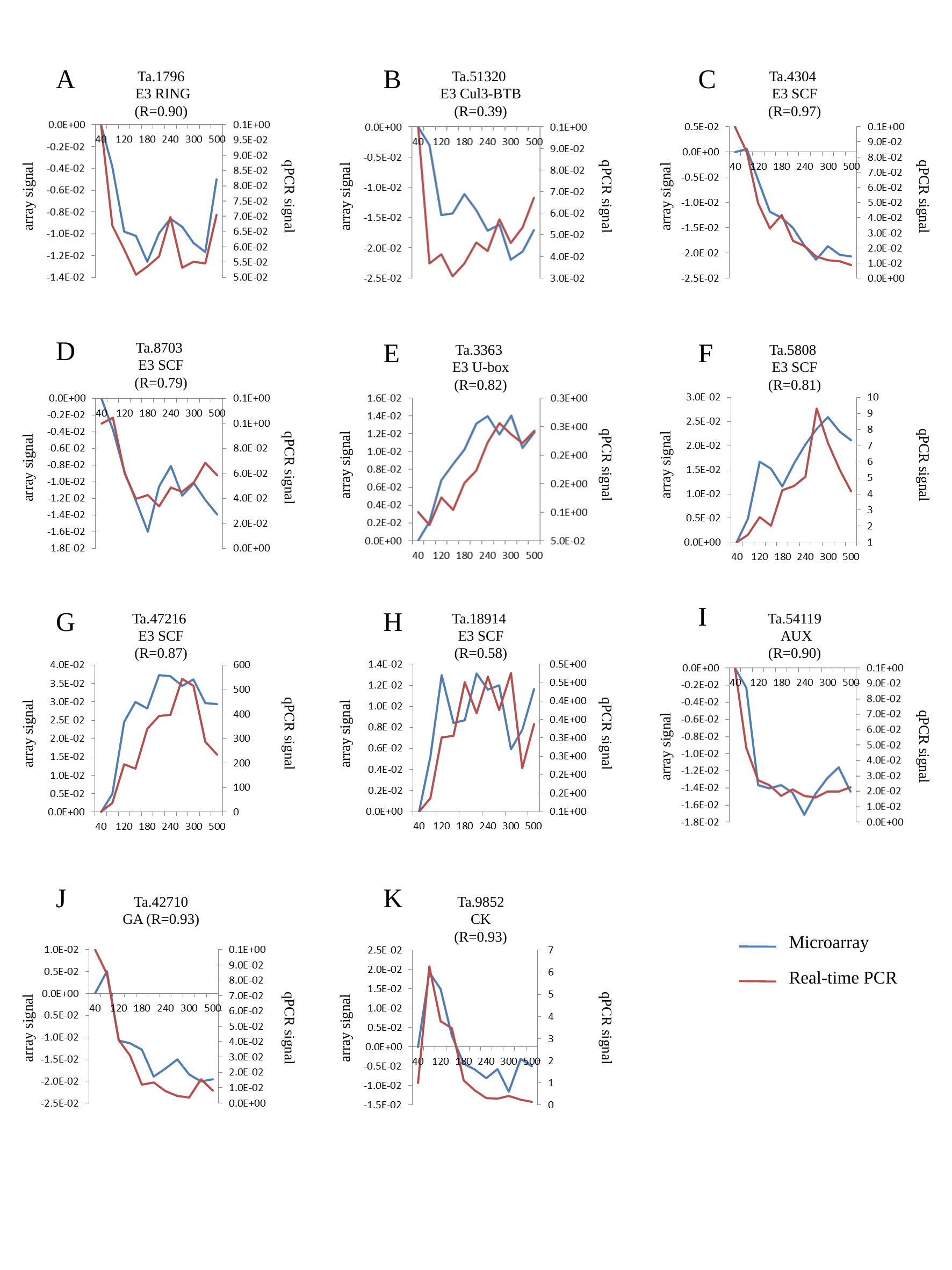

A
B
C
Ta.1796
 E3 RING (R=0.90)
Ta.51320
E3 Cul3-BTB (R=0.39)
Ta.4304
E3 SCF (R=0.97)
array signal
array signal
array signal
qPCR signal
qPCR signal
qPCR signal
D
E
F
Ta.8703
E3 SCF (R=0.79)
Ta.3363
E3 U-box (R=0.82)
Ta.5808
E3 SCF (R=0.81)
array signal
array signal
qPCR signal
qPCR signal
array signal
qPCR signal
I
G
H
Ta.47216
E3 SCF (R=0.87)
Ta.18914
E3 SCF (R=0.58)
Ta.54119
 AUX (R=0.90)
array signal
qPCR signal
array signal
qPCR signal
array signal
qPCR signal
J
K
Ta.42710
GA (R=0.93)
Ta.9852
CK (R=0.93)
Microarray
Real-time PCR
array signal
array signal
qPCR signal
qPCR signal
